# Impact of hospital lead extraction volume on management of cardiac implantable electronic device-associated infective endocarditis: does size really matter?

**DOI:** 10.1093/europace/euae307

**Published:** 2024-12-24

**Authors:** Sandra Howell, Alphonsus Lieuw, Christopher Aldo Rinaldi

**Affiliations:** Department of Imaging Sciences, King's College London, St Thomas's Hospital, London SE1 7EH, UK; Department of Imaging Sciences, King's College London, St Thomas's Hospital, London SE1 7EH, UK; Department of Imaging Sciences, King's College London, St Thomas's Hospital, London SE1 7EH, UK; Department of Cardiology, Guy's and St Thomas' Hospitals, London SE1 7EH, UK; Heart Vascular and Thoracic Institute, Cleveland Clinic London, 33 Grosvenor Place, London SW1X 7HY, UK

The increase in number of cardiac implantable electronic device (CIED) implants in patients with increasing age and comorbidities is paralleled by an increasing risk of infection and endocarditis.^1^ CIED infection is associated with a high morbidity and mortality with transvenous lead extraction (TLE) significantly reducing mortality, especially if performed at an early stage.^1^ Despite TLE being a Class I recommendation in the setting of cardiac implantable electronic device associated infective endocarditis (CIED IE), only a minority of patients with device infections receive TLE.^2^ Sciria *et al*.^2^ using hospital admissions data from the US healthcare system between 2016 and 2019 demonstrated that only 11.5% of patients with CIED IE underwent TLE, but in those patients receiving TLE there was a significant reduction in mortality (6% vs. 9.5%, *P* < 0.05). Certain factors, including *Staphylococcus aureus* infection, presence of an implantable cardiac defibrillator (ICD), and large hospital size, were associated with increased rates of TLE, whereas it was less likely to be undertaken in older and female patients as well as those with dementia and chronic kidney disease.TLE has been demonstrated to have excellent procedural success and safety data.^3–5^ The ELECTRa study included over 3500 patients in 73 centres in 19 European countries undergoing lead extraction with a procedural success rate of over 96% and a low rate of complications with a major procedural complication rate of 1.7% and a procedure-related death rate of 0.5%.^3^ In ELECTRa extraction centres were dichotomized into low- and high-volume centres on the basis of an annual procedural volume of 30 TLE procedures per year and demonstrated better outcomes and lower mortality in high-volume extraction centres. Sidhu *et al*.^6^ subsequently demonstrated that outcomes following extraction, including mortality were superior if undertaken in high-volume centres.

CIED infection/IE is therefore associated with a poor prognosis and has an effective treatment (TLE) which if undertaken in a timely manner significantly reduces mortality, but despite a high level of recommendation, the uptake of TLE in CIED IE remains low. An understanding of the barriers that may prevent patients from undergoing TLE is critical. The current study by Mandler *et al*.^7^ in this journal is therefore apposite in examining the impact of hospital TLE volume on the management of CIED-associated IE. The authors expand on their previous findings^2^ by examining the impact of hospital TLE procedural volume on TLE utilization and outcomes for 21 545 admissions with CIED-associated IE using the Nationwide Readmissions Database between 2016 and 2019. TLE centres were categorized based on annual volume tertiles into low-volume centres (1–17), medium-volume centres (18–45), and high-volume centres (>45 TLEs/year). The majority (57%) of admissions were to low-volume centres with TLE performed in only 6.9, 19.3, and 26% of admissions at low-, medium-, and high-volume centres, respectively (*P* < 0.001). Furthermore, among patients with *S*. *aureus* infections, TLE was performed in 16.4, 36.6, and 41.4% of admissions to low-, medium-, and high-volume TLE centres, respectively (*P* < 0.001). After adjustment for age and comorbidities, hospitalization for IE at high-volume centres was independently associated with TLE compared with low-volume centres (OR 4.26). Importantly, across each subgroup of patients at low-, medium-, and high-volume centres TLE utilization was associated with a significantly lower mortality. The authors also found that LE-associated complication rates were similar at low-, medium-, and high-volume centres (2.5, 2.3, and 3.4%, respectively, *P* = 0.493) as was inpatient mortality. The authors conclude there is higher utilization of TLE for CIED-associated IE in high-volume centres compared with lower volume centres. The authors state that although TLE-associated complications and mortality were similar when stratified by hospital TLE volume differences, this finding may be explained by differences in patient comorbidities and complexity between centres. The issue of comorbidities is important with patients at low volume TLE centres more likely to be older and female with a higher prevalence of chronic lung disease and dementia, whereas patients at high-volume centres had more ICDs, obesity, congestive heart failure, cerebrovascular disease, chronic liver disease, coagulopathy, drug abuse, and *S. aureus* infection.

The authors are to be commended for this timely analysis, which exposes some of the barriers to TLE in patients with IE. TLE for IE is more likely to be undertaken in high-volume centres, especially in patients with *S. aureus* infections. There are, however, several limitations in relation to the findings of outcomes in differing volume centres that the authors rightly highlight. This is a retrospective analysis covering only 50% of hospitals in the United States, which affects the generalizability of the findings, and the study relies on ICD-10-CM coding with the inherent problem of missing or inappropriate coding. Although total hospital extraction volume is measured, there is no operator-level data, and moreover, ICD-10-CM codes may not distinguish between transvenous and leadless/subcutaneous devices, and therefore a subset of patients may not have had transvenous leads available for extraction. In terms of TLE-associated complications, these may have been over-estimated as ICD-10-CM codes do not assign causality to the procedures performed thus patients might have had concurrent valve surgery, which could account for complications. Other important factors that may affect outcomes, including time from initial diagnosis to TLE, were not available, which is a particular confounder, as outcomes are worse when there is a delay to TLE in the setting of sepsis. Probably most importantly, from the procedural complication standpoint, lead dwell times were not available, and it is well established that longer dwell times (>10 years) are predictive of procedural complications and death.^3^ These patients with prolonged dwell times are typically managed by high-volume centres, and therefore complications and mortality associated with TLE across differing volume centres in this study should be interpreted with caution. Finally, mortality data for this study were only in-hospital and out-of-hospital deaths were not included.

Although the current study by Mandler *et al*. has limitations, as acknowledged by the authors, it does provide valuable insight into the practice of TLE for CIED IE and confirms previous findings that the majority of patients hospitalized with CIED IE do not receive guideline-recommended TLE. The findings support the use of TLE being undertaken without significant delay in an appropriate environment. Lack of risk adjustment for critical procedural factors, including implant duration, limits the conclusions related to complication rates and mortality in centres of differing volume. The current study is therefore limited in its ability to define whether these procedures should be undertaken in lower volume centres, but importantly, hospitalization in a large volume TLE centre was associated with a greater chance of undergoing TLE, which would support referral of such patients to high-volume centres. There is clearly still a lot of work to be done to explore the reasons for the low uptake of lead extraction and the barriers that prevent many of these patients from receiving life-saving treatment.
